# An Approach to Self-Assessed Auditory Wellness in Older Adults

**DOI:** 10.1097/AUD.0000000000001001

**Published:** 2021-03-09

**Authors:** Larry E. Humes

**Affiliations:** Department of Speech, Language, and Hearing Sciences Indiana University Bloomington, IN, USA

*Editor’s Note: The following article is based on the 2020 American Auditory Society Carhart Memorial Lecture and is categorized as a “Point of View” article. As originally described (Jerger 2000): “Our second type of new publication, the Point of View article, is a publication with a slant or opinion. This type of article should have a fresh point of view, a clear logic to its propositions, and a clarity of presentation. The article must have a well-reasoned point of view, but the view does not have to be balanced. Our long-term goal for the Point of View article is to stimulate the field’s interest in and to enhance the appreciation of the author’s area of expertise.”*

In 2008, under Amy Donahue’s leadership, the National Institute on Deafness and other Communication Disorders (NIDCD) convened the Working Group on Accessibility and Affordability of Hearing Healthcare. A summary of that group’s deliberations and recommendations appeared in this journal a decade ago (Donahue et al. 2010). The purpose of the working group was to address a long-standing problem: most adults with mild-to-moderate hearing loss who could potentially benefit from hearing aids were not obtaining them. Before the meeting of the NIDCD Working Group, both industry surveys and epidemiological studies had documented that only about 15 to 30% of adults with mild-to-moderate hearing loss were obtaining hearing aids (Sangster et al. 1991; Kochkin 1993a,b,c; Ward et al. 1993; Popelka et al. 1998; Kochkin 2000; Smeeth et al. 2002; Dalton et al. 2003; Chia et al. 2007; Kochkin 2009) and this has remained the case since (Wilson et al. 2010; Chien & Lin 2012; Dawes et al. 2014; Bisgaard & Ruf 2017). The general problem underlying the limited uptake of hearing aids was believed to be the poor affordability and accessibility of hearing healthcare, especially treatment via hearing aids (Donahue et al. 2010).

Moreover, the failure of the prevailing service-delivery model at the time meant that millions of adults with untreated hearing loss would continue to suffer broader consequences. It is well-known, for example, that the loss of audibility alone causes many difficulties for everyday speech communication, including poor speech perception (e.g., Humes & Dubno 2010) and increased listening effort (e.g., Pichora-Fuller et al. 2016), among others. Furthermore, it has been long established that untreated hearing loss can lead to a variety of psychosocial problems, with most studies focusing on depression (e.g., Cosh et al. 2019). Finally, there is mounting evidence that untreated hearing loss can have a negative impact on cognitive function and that hearing aids *may* help to reduce that impact (e.g., Amieva & Ouvrard 2020). In summary, the poor uptake of hearing aids was a serious problem with broad potential consequences on everyday function and well-being.

The NIDCD Working Group’s activity and the summary report of Donahue et al. (2010) provided the impetus for a series of important events leading to an envisioned world with improved accessibility and affordability of hearing aids. Critical to this vision was the empowerment of the adult with mild-to-moderate hearing loss to evaluate their own hearing difficulties and to seek solutions directly without the assistance of a healthcare professional. The President’s Council of Advisors on Science and Technology (PCAST) issued a report endorsing this self-directed path to hearing aids, advocating for the creation of Over-the-Counter (OTC) hearing aids in particular (PCAST 2015). This was followed by a similar recommendation in a report by the National Academies of Sciences, Engineering, and Medicine (NASEM 2016). Ultimately, this led to the US Federal OTC Hearing Aid Act of 2017 which was signed into law in 2018. The US Food and Drug Administration is expected to release draft rules and guidelines for OTC hearing aids in 2021.

Two key points pertaining to the 2017 OTC Hearing Aid Act are especially noteworthy. First, these devices are “…intended to be used…to compensate for *perceived* mild to moderate hearing impairment…” in adults. There is no requirement for audiometry to substantiate the presence of a hearing loss. Rather, it relies on the adult’s perception of hearing difficulties. Second, the Act also notes that “…tests for self-assessment of hearing loss…” *may* be used but are not required.

Clearly, a new era is dawning for the way in which hearing aids will be delivered to many adults with perceived mild-to-moderate hearing loss. It is important to recognize that this new era is not driven exclusively by the OTC Hearing Aid Act. Rather, the Act is a product of a much broader initiative to revamp healthcare from a medical model of treating illness after it occurs to a preventative public-health model of individuals empowered to manage their own health and wellness proactively (e.g., Johnson 2011; Hibbard 2017; Higgins et al. 2017). So many modifiable risk factors have been identified that contribute to age-related hearing loss that some consider it to be a largely preventable disorder (e.g., Cruickshanks et al. 2010; Nash et al. 2011). Self-awareness of declining auditory wellness and its consequences may motivate the older adult to minimize such risk factors. Nonetheless, age-related hearing loss is not entirely preventable and intervention following declining auditory wellness is often needed. More accessible and affordable options for hearing healthcare, including the acquisition of OTC hearing aids, are envisioned as important steps in the ability of older adults to manage their own auditory wellness. Broad access to self-assessment tools for auditory wellness, as well as for the evaluation of interventions, is critical for the success of a preventative wellness model. Moreover, the range of interventions must not be confined to the access of devices alone but should include recommendations for other efficacious interventions, such as communication training (e.g., Hickson et al. 2007), when appropriate.

Unquestionably, the largest group of adults with documented mild-to-moderate hearing loss is those over the age of 50 years (e.g., Cruickshanks et al. 2010; Lin et al. 2011). Although not explicitly stated in the OTC Hearing Aid Act, it can be presumed that it was designed with this age group in mind because this was clearly the emphasis in prior reports leading up to the adoption of the Act (PCAST 2015; NASEM 2016). With this assumption in mind, are there existing tools that can be used to guide the older adult in establishing reliably and validly that they have *perceived* hearing difficulties that warrant pursuit of an OTC hearing aid? In the pages that follow, it is suggested that the Hearing Handicap Inventory for the Elderly (HHIE), either the full 25-item inventory or (Ventry & Weinstein 1982; Weinstein & Ventry 1983) the 10-item screener (Ventry & Weinstein 1983; Weinstein 1986), could be an excellent tool to guide older adults in the self-assessment and management of their auditory wellness.

Defining auditory wellness from a self-report measure such as the HHIE is superior to reliance on pure-tone audiometry in the context of the international model of healthy function established by the World Health Organization (WHO 2001). This conceptual framework of an individual’s function and health gave rise to the now-commonplace WHO International Classification of Functioning, Disability and Health (ICF). It is not the actual hearing-impairment classification system that is of interest here as this has been reviewed recently elsewhere (Humes 2018, 2019a,b). Nor is it the application of this model to audiology as this also has been reviewed in detail elsewhere (Meyer et al. 2016; Humes et al. 2020) and there are ongoing activities to enhance this application (e.g., Granberg et al. 2014a,b; https://icra-audiology.org/documentsworkinggroups/icf-core-set-for-hearing-loss). Rather the focus here is even broader: on the key components of the conceptual model of individual health and wellness identified in the WHO-ICF model.

In the WHO-ICF model, the individual’s health and well-being are determined by the status of the person’s: (1) body functions and structures; (2) daily activities; and (3) participation in the world around them. Full healthy function has few bodily impairments, limitations on daily activities, and restrictions on participation in the society. Conversely, declines in wellness, sometimes referred to previously as disabilities and handicaps, result from varying deficits in any or all three key personal factors. Importantly, it is recognized in the model that wellness is not determined solely by these three factors, but in a context of: (1) environmental factors; and (2) personal factors. These contextual factors modulate the impact of any deficits in the three primary components of wellness and the modulation may have either a positive or negative impact on wellness. Pure-tone audiometry, and other measures strongly correlated with it such as speech-recognition threshold, captures only one of the three key primary factors of auditory wellness: impairment of bodily functions or structures. Well-conceived self-report measures, on the other hand, have the potential to capture function in all three personal domains and may also encompass the modulating environmental and personal contextual factors.

In the WHO-ICF model, excellent wellness in any domain would be characterized by healthy, unimpaired bodily structures and functions, full execution of daily tasks or activities (i.e., no activity limitations), and unrestricted participation in life events and situations (i.e., no participation restrictions), as well as an absence of environmental or personal barriers limiting function. For a given or standardized set of contextual factors, excellent auditory wellness is conceptualized here as excellent auditory functioning or no auditory disability. That is, there are no impairments to auditory structures and functions, no limitations imposed on daily activities due to poor auditory function, and no restrictions in participation resulting from auditory difficulties. On the other hand, in this same standardized context, very poor auditory wellness is considered equivalent to very poor auditory functioning or severe auditory disability. Perceived auditory wellness is considered here to be inversely related to perceived auditory disability or handicap. As a result, well-conceived self-report measures of perceived auditory disability or handicap offer indirect measures of auditory wellness.

The HHIE represents just such a well-conceived self-report measure of auditory disability and handicap for older adults. It was designed to be sensitive to the consequences of measured pure-tone hearing loss while capturing hearing difficulties impacting social function and emotional well-being in response to those difficulties. The HHIE has a special focus on older adults, generally 65 and over (Ventry & Weinstein 1982; Weinstein & Ventry 1983).

The full HHIE, referred to here as the HHIE-Total, consists of 25 items, 12 that tap social aspects of hearing difficulties (e.g., “Does a hearing problem cause you to attend religious services less often than you would like?”) and 13 that assess the emotional reactions to hearing difficulties (e.g., “Does a hearing problem cause you to feel embarrassed when meeting new people?”). Response choices and point values are: yes (4 points); sometimes (2 points); and no (0 points). This results in a range of possible HHIE-Total scores of 0 (no difficulties) to 100 (severe difficulties). Although the 25 HHIE items have sometimes been analyzed separately as 12-item HHIE-Social and 13-item HHIE-Emotional subtests, research does not support doing so (e.g., Noble et al. 2008; Cassarly et al. 2020). In addition to the full 25-item HHIE, a brief 10-item screener was developed and evaluated by Ventry and Weinstein (1983). The response options and assigned points remain the same which results in a range of possible scores for the HHIE-S from 0 (no problems) to 40 (severe difficulties).

Although the HHIE was extended to adults under age 65 by making slight modifications to form the Hearing Handicap Inventory for Adults (HHIA; Newman et al. 1990), there is a much larger volume of data available for evaluation of the HHIE. In addition, as a part of the dataset from the Medical University of South Carolina (MUSC) described below, 160 older adults had completed both the full HHIE and HHIA self-report measures. The correlation (*r* = 0.995, *p* < 0.001) and best-fitting linear regression [HHIE = 0.9 + 0.95 × HHIA, *F*(1,158) = 16,168, *p* < 0.001] for these data support that these two measures are interchangeable and they will be treated as such here. Owing to the largest volume of data existing for the screening version of the HHIE, the HHIE-S, the initial focus is placed on the HHIE-S for the development and evaluation of a proposed scale of auditory wellness. Once these voluminous data have been reviewed and a wellness scale proposed, the scale will be translated to one based on the full 25-item HHIE-Total score. This translation facilitates further validation of the proposed auditory wellness scale. After evaluation of this proposed auditory-wellness scale, the recent work of Cassarly et al. (2020) on the Revised Hearing Handicap Inventory (RHHI), derived from both the HHIE and HHIA, will be reviewed. This paper concludes with a consideration of the RHHI as another HHIE-based option for a self-report measure of perceived auditory wellness.

## EVALUATION OF THE HHIE-S

Table 1 summarizes several demographic characteristics of the key datasets used in the evaluation of the HHIE-S as a potential measure of auditory wellness. Three datasets, the MUSC convenience sample of the Charleston, South Carolina area (Cassarly et al. 2020), the Blue Mountains (BM) Australian population study (Sindhusake et al. 2001), and the Epidemiology of Hearing Loss Study (EHLS), a population study of Beaver Dam, Wisconsin (Wiley et al. 2000), represent broad samples of generally nonclinical populations. These data are pooled to represent normative or nonclinical distributions of the HHIE-S scores among the general population of adults 50 years of age or older. Two datasets, the Veterans Administration (VA) clinical dataset from Eastern Tennessee, much of which was published in Wilson (2011), and the Mayo Clinic (Mayo) clinical dataset from Jacksonville, Florida, an unpublished dataset, were combined to generate representative distributions of HHIE-S scores for adults over the age of 50 who reported to the clinic for help. When all five datasets are pooled, the total N is 12,571. Table 1 provides additional details about each of these datasets.

**TABLE 1.** Demographics of the datasets used in various analyses in this article

Dataset			PTA4		Age (yr)		HHIE/A-S	
	N	%Female	M	SD	M	SD	M	SD
Normative/population								
Blue Mountains	2843	56.9	22.0	14.6	67.4	9.2	7.4	8.9
EHLS*	1567	0.0	23.0	65.8				
MUSC	1190	57.6	25.6	14.4	69.4	7.4	9.9	9.4
Clinical								
Mayo	4584	38.3	30.6	15.8	70.2	10.7	16.4	11.4
VA	2387	0.0	37.4	12.7	65.3	8.9	25.8	9.9

Ages were > 50 years, except for the ELHS data which ranged from 48 to 89 years. PTA4 is the pure-tone average for 500, 1000, 2000, and 4000 Hz in the better ear.

HHIE/A-S refers to the 10-item screening version of either the HHIE or HHIA, whichever was used in that study. Only the Mayo dataset is exclusively from the HHIA-S.

* The EHLS HHIE-S data were extracted from Figure 9 of Wilson et al. (2010) and only partial demographic information was available in that article for this subset of male Veterans and non-Veterans of the EHLS1 cohort.

EHLS, epidemiology of hearing loss study; HHIA, Hearing Handicap Inventory for Adults; HHIE, Hearing Handicap Inventory for the Elderly; MUSC, Medical University of South Carolina; VA, veterans affairs (East Tennessee Region).

published online ahead of print March 9, 2021.

Although the five datasets in Table 1 have been dichotomized into “normative” and “clinical” samples, the means and standard deviations for the PTA4 values (average pure-tone threshold for 500, 1000, 2000, and 4000 Hz) indicate that there is overlap in the amount of hearing loss across these two categories. The primary basis for dichotomization of these five studies into two groups was not the audiometric characteristics of the samples but the nature of the sample. As noted, the normative studies represent broad samples of the local community, whereas the clinical studies were datasets drawn from clinic patients who sought professional evaluation of their hearing difficulties. It is assumed here that the overwhelming majority of those in the clinical samples had perceived difficulties sufficient to warrant a visit to the clinic, whereas this was not the case for most of those in the normative studies. In support of this conjecture, data for the three normative studies indicate that only 7.4 to 14.6% of the population or community samples were currently or usually using hearing aids (Popelka et al. 1998; Chia et al. 2007) with a visit to the clinic required to obtain the hearing aids. Although it is likely that others in the normative samples aside from those currently using hearing aids had visited the clinic, these data on the prevalence of hearing aid use in the normative samples suggest that it was a minority who did so. Clearly, 100% of those in the clinic samples went to the clinic for evaluation, although it is unclear whether the visit was entirely self-motivated by perceived difficulties or driven by other factors. The prevalence of hearing aid use among the clinic samples is also unknown but assumed to be considerably greater than that of the population samples. In addition to these considerations, as noted in Table 1, the combined clinical dataset is biased toward males. Furthermore, for the samples in Table 1, only 15% of the older adults were in their 80s and, overall, more than 95% were non-Hispanic Whites. These data limitations should be kept in mind when attempting to broadly apply the auditory wellness measures proposed here.

**Fig. 1.**
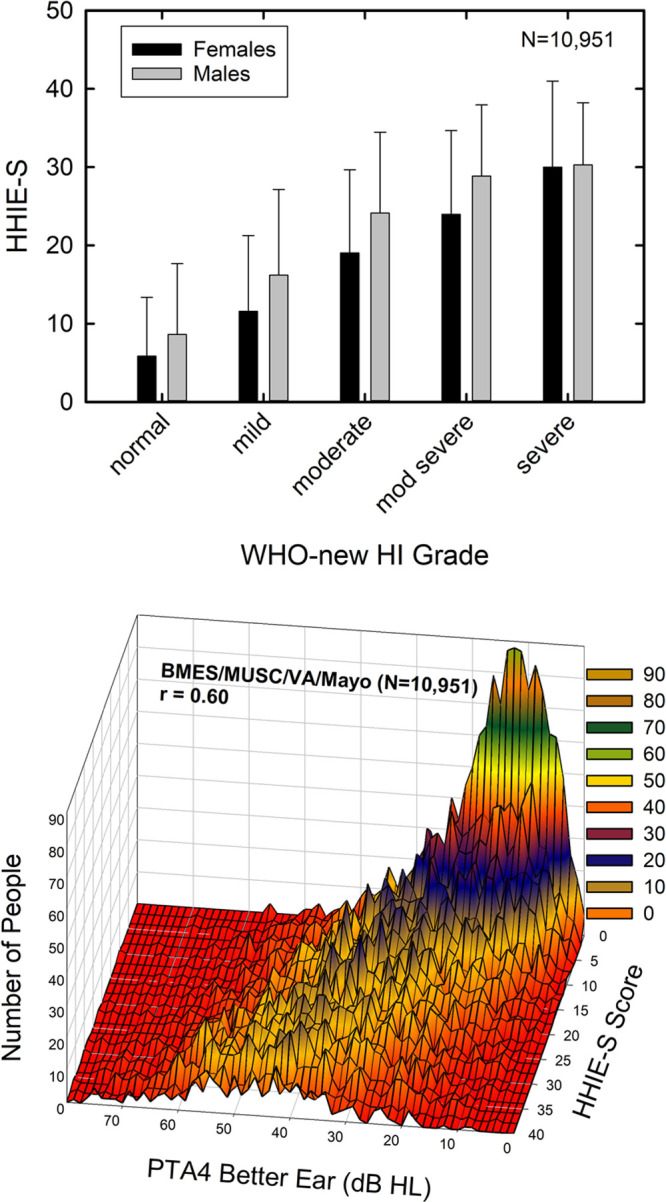
The top panel shows mean HHIE-S scores for the pooled dataset (N = 10,951) when grouped by gender (females, black bars; males, gray bars) and WHO-new hearing-impairment grade (Stevens et al. 2013). Error bars represent 1 standard deviation above the mean. The bottom panel shows the same HHIE-S data as the top panel but as a contour plot with better-ear PTA4 in dB HL along the X-axis. The color scale represents the number of individuals counted with each PTA4/HHIE-S combination. HHIE-S, Hearing Handicap Inventory for the Elderly-Screener.

The HHIE-S captures the sensory-impairment component of auditory wellness as demonstrated by the group data in the top panel of Figure 1. The bar graph in the top panel shows the means and SDs for HHIE-S scores when the data were pooled for four of the five datasets listed in Table 1 (N = 10,951). (It was not possible to obtain access to the raw data for the EHLS dataset for these analyses and they were not included here as a result.) The HHIE-S scores are plotted as a function of WHO-new Hearing-Impairment (WHO-new HI) grade (Stevens et al. 2013) in the top panel of Figure 1. For the WHO-new HI grade, better-ear PTA4 ≤ 20 dB HL corresponds to “normal,” 21 to 35 dB HL to “mild,” 36 to 50 dB HL to “moderate,” 51 to 65 dB HL to “moderately severe,” 66 to 80 dB HL to “severe,” and >80 dB HL to “profound” hearing impairment. There appears to be an orderly progression of perceived difficulty, as measured by the HHIE-S, with the severity of the measured pure-tone hearing loss in both men (gray bars) and women (black bars). However, as the contour plot in the lower panel of Figure 1 reveals, there is a wide range of perceived hearing difficulties for a given better-ear PTA4; the measure used to generate the WHO-new HI grade in the top panel. For example, consider the case of the better-ear PTA4 = 20 dB HL, the upper limit for the “normal” WHO-new HI grade. Although most individuals with a better-ear PTA4 of 20 dB HL have low HHIE-S scores of 0 to 10, many others with that same PTA4 have HHIE-S scores ranging from 10 to 40. As noted in the lower panel of Figure 1, the Pearson-r correlation between the HHIE-S score and the better-ear PTA4 for the combined dataset was 0.60 (*p* < 0.001). Thus, HHIE-S is sensitive to the degree of sensory impairment with shared variance (*r*^2^) of 36% between these two measures. Importantly, however, PTA4 can only explain about half of the systematic variance in HHIE-S scores. That is, assuming test–retest reliability of *r* = 0.9, as noted below, 81% (*r*^2^) of the variance is considered systematic and 44% of the systematic variance (0.36/0.81) is explained by PTA4. The correlation between age and HHIE-S scores for this same combined dataset was *r* = 0.12 (*p* < 0.001) and the partial correlation was *r*_p_ = −0.22 (*p* < 0.001) with better-ear PTA4 as a covariate. Although both correlations are significant, age alone can only account for another 1 to 4% (*r*^2^ = 0.12^2^ to 0.22^2^ or 1 to 4%) of the variance in HHIE-S scores. These data support the notion that the HHIE-S captures the impact of sensory impairment but is also capturing other aspects of the individual’s perceived hearing difficulties not related to hearing loss.

**Fig. 2.**
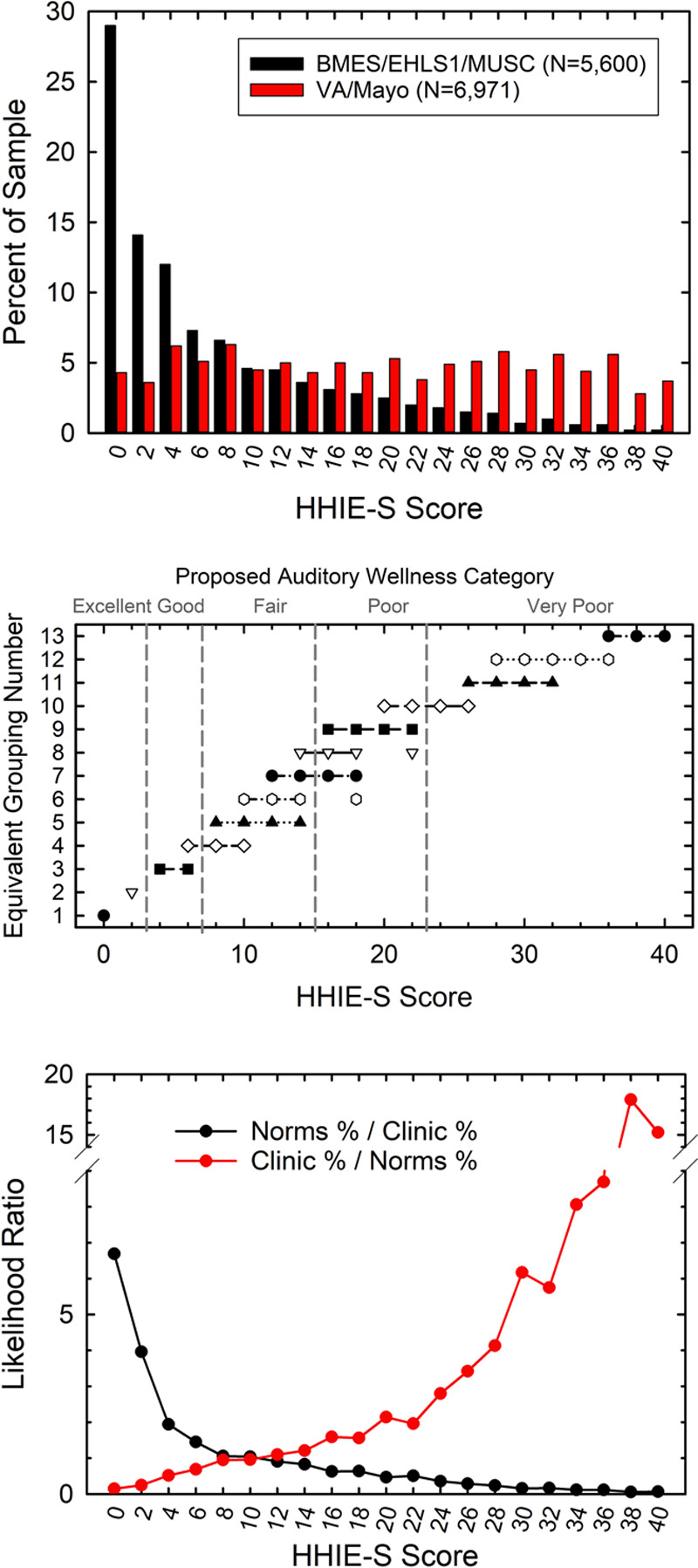
The top panel shows the observed percentages of individuals within the normative population sample (black bars) and the clinical sample (red bars) who had each of the possible HHIE-S scores. The middle panel shows groups of HHIE-S scores for which the dataset proportions do not differ significantly from one another based on z-tests of the proportions. For example, for an HHIE-S score of 0, the normative and clinical proportions in the top panel differed from all other HHIE-S-score proportions resulting in a single score, 0, comprising Group 1. The bottom panel shows the likelihood ratios calculated from the observed percentages in the top panel, either favoring the normative sample (black circles and lines) or the clinical sample (red circles and lines). HHIE-S, Hearing Handicap Inventory for the Elderly-Screener.

The normative nonclinical and clinical datasets summarized in Table 1 were used to establish representative distributions of HHIE-S scores in these two types of populations. The normative nonclinical HHIE-S scores illustrate the range of perceived hearing difficulties in the general population of adults 50 years of age and older. The clinical datasets, on the other hand, depict the distribution of HHIE-S scores for those who are presumed to have felt that their difficulties were sufficiently severe to seek help at the clinic. In the top panel of Figure 2, the distributions of observed HHIE-S scores spanning the full range from 0 to 40 are shown for each dataset type. Whereas the clinical datasets show a fairly even distribution of scores from 0 to 40, the nonclinical normative datasets show a skewed distribution with over half of the individuals reporting little or no perceived difficulties (scores from 0 to 4).

An omnibus Chi-square analysis of the clinical and normative distributions in the top panel of Figure 2 found that the distributions differed significantly [X^2^(20) = 3309.5, *p* < 0.001]. This significant difference in distributions supports several of the assumptions described above about the likely differences in perceived hearing difficulties between those comprising the clinical and normative datasets. That is, many in the normative sample perceived no hearing difficulty, whereas most in the clinical samples perceive considerable hearing difficulties as measured by the HHIE-S.

Follow-up Bonferroni-adjusted (*p* < 0.05) z-tests of proportions were used to compare the proportions for a given HHIE-S score to the proportions for all other scores. Given 21 possible HHIE-S scores, even numbers from 0 to 40, this involved 210 paired comparisons [(21 × 20)/2] of proportions. Groups of scores with nonsignificantly different proportions are connected by horizontal lines in the middle panel of Figure 2. Conceptually, separate symbols and horizontal lines in the middle panel identify groups of scores with equivalent proportions for both samples across the range of possible HHIE-S scores. For example, the proportions for the HHIE-S score of 0 differed significantly from all other HHIE-S score proportions and this constituted the first HHIE-S score group (filled circle, lower left). Likewise, the proportions for the HHIE-S score of 2 in the top panel differed significantly from all other HHIE-S score proportions and constituted the second grouping of HHIE-S scores (unfilled inverted triangle, lower left). Next, the proportions for the HHIE-S scores of 4 and 6 did not differ from one another (filled squares connected by horizontal line) but differed from all other lower and higher score groupings. Each subsequent grouping of equivalent HHIE-S score proportions is identified by separate symbols connected by horizontal lines. Progressing to the far right (middle panel, top right), the proportions for HHIE-S scores of 36, 38, and 40 did not differ significantly from one another but did differ significantly from all other score groupings. All told, 13 “equivalent proportion” groupings were identified via the z-tests of the proportions as identified in the middle panel of Figure 2.

Next, from the distributions of observed HHIE-S scores for the two datasets in the top panel of Figure 2, likelihood ratios were generated in favor of the score coming from either the normative dataset (norms %/clinic %) or the clinical dataset (clinic %/norms %). The calculated likelihood ratios appear in the bottom panel of Figure 2. With these likelihood ratios in hand, consider an older adult who obtains an HHIE-S score of 2. Clearly, it is more likely that this individual represents someone from the general nonclinical population who perceives little hearing difficulty. The middle panel also indicates that individuals with a score of 2 are independent of those with all other possible HHIE-S scores. On the other hand, given the likelihood ratios in the bottom panel, it is much more likely that a score of 24 or higher on the HHIE-S is from a clinical sample representative of those experiencing enough difficulty to have sought help at the clinic. In addition, as shown in the middle panel, those with HHIE-S ≥ 24 are largely independent of those score groupings with lower scores. Based on the equivalent-proportion groupings in the middle panel and the distributions of likelihood ratios in the bottom panel of Figure 2, five categories of auditory wellness were approximated visually and designated: (1) “Excellent” auditory wellness or no hearing difficulties, HHIE-S scores of 0 or 2 and likelihood ratio > 4 in favor of the normative sample (bottom panel) and little overlap of these scores with other HHIE-S scores (middle panel); (2) “Good” auditory wellness with few hearing difficulties, HHIE-S scores of 4 or 6 and likelihood ratio >1 in favor of the normative sample and little overlap with other HHIE-S scores; (3) “Fair” auditory wellness, HHIE-S scores of 8 to 14, with roughly equivalent likelihood ratios (about 1.0) for the normative and clinical samples and some overlap with adjacent wellness categories; (4) “Poor” auditory wellness, HHIE-S scores of 16 to 22, and likelihood ratios > 1 in favor of the clinical samples and some overlap with adjacent categories; and (5) “Very Poor” auditory wellness, HHIE-S scores from 24 to 40 and likelihood ratios in favor of the clinical dataset >3.0 and little overlap of these scores with all other HHIE-S scores.

Although the proposed five-category scale of auditory wellness was driven by consideration of the patterns of results in the middle and bottom panels of Figure 2, the choice of the number of categories was somewhat arbitrary. For example, the lower two panels of Figure 2 would support finer-grained wellness categories at both ends of the HHIE-S scale with perhaps as many as seven total categories (two each within the proposed “excellent” and “very poor” categories). On the other hand, one could argue that there is considerable overlap among the proposed “good” and “fair” categories such that only one broad range of HHIE-S scores from 8-26 is needed for “fair.” To further evaluate the use of five categories, the pooled data from the normative and clinical datasets were evaluated using a two-step cluster analysis of the HHIE-S scores. Up to 15 clusters were possible but the analysis yielded five clusters as the best fit and it was a good fit based on the Bayesian Information Criterion.

Finally, there was some consideration given to the nature of the response patterns within each wellness category. For example, to obtain the scores of 0 or 2 for an auditory wellness rating of “excellent,” the respondent replied either “No” to all 10 items or to nine of 10 items plus “Sometimes” to the remaining item. No “Yes” responses are possible for this wellness rating. To progress to the next wellness rating of “good,” the possible responses included either 1 “Yes” and 1 “Sometimes” or 3 “Sometimes.” This transition from no “Yes” responses to at least 10% “Yes” or 30% “Sometimes” was considered a meaningful transition. Likewise, for auditory wellness to be rated “fair,” the possible responses ranged from 20 to 30% “Yes” to 70% “Sometimes.” For a wellness rating of “poor,” possible responses ranged from 40 to 50% “Yes” to 10% “Yes” with 90% “Sometimes.” For the worst auditory wellness, a rating of “very poor,” response possibilities range from 60 to 100% “Yes” responses to a mix of 20% “Yes” plus 80% “Sometimes” responses. Each of the ranges of response patterns for successive steps along the auditory wellness rating seemed to be meaningful changes in perceived difficulties to the author, but the subjectivity of this assessment is acknowledged.

In summary, it is concluded that a five-point scale is a reasonable first approximation for the proposed scale of auditory wellness. In the sections that follow, the proposed five-category rating will be validated. However, this does not mean that other scales of auditory wellness formed from the same or similar datasets would be invalid. Further research is required to evaluate the most appropriate scale of auditory wellness, including the number of categories or ratings of auditory wellness needed as well as the cut points for those categories.

From the inception of the abbreviated HHIE-S, the goal was to have a measure that related to the measured pure-tone hearing loss but captured more than the sensory impairment alone (Ventry & Weinstein 1983). Ventry and Weinstein (1983) suggested that HHIE-S scores of 0 to 8 represented “no self-perceived handicap,” 10 to 22 “mild to moderate handicap,” and 24 to 40 “significant handicap.” Others have since validated the use of very similar cut points for screening purposes in other clinical samples (e.g., Lichtenstein et al. 1988; Sindhusake et al. 2001; Wilson et al. 2010; Tomioka et al. 2013). These cut points for use of the HHIE-S as a screen for impaired hearing are very close to those at the boundaries between “good”/”fair” and “poor”/”very poor” auditory wellness ratings. In all these studies, the HHIE-S results were evaluated against the measured pure-tone thresholds in the same individuals as the latter was considered the gold-standard for severity of hearing difficulties. In the case of auditory wellness, however, the objective is not to attain good agreement with measured pure-tone thresholds, as was the case in prior evaluations of the HHIE-S. Rather, the HHIE-S is considered “the gold standard” for auditory wellness and pure-tone thresholds contribute partially to that measure of perceived wellness. In that regard, it should also be noted that the HHIE-S is framed more as a measure of perceived hearing difficulties or negative consequences of those difficulties. As a result, low scores imply few perceived difficulties which, in turn, are assumed here to imply “excellent” or “good” auditory wellness. Ideally, direct measures of auditory wellness will be developed for future applications. In the interim, the vast amount of data currently available for the HHIE-S, a measure of perceived difficulties, is inverted to capture perceived wellness rather than “handicap.”

**Fig. 3.**
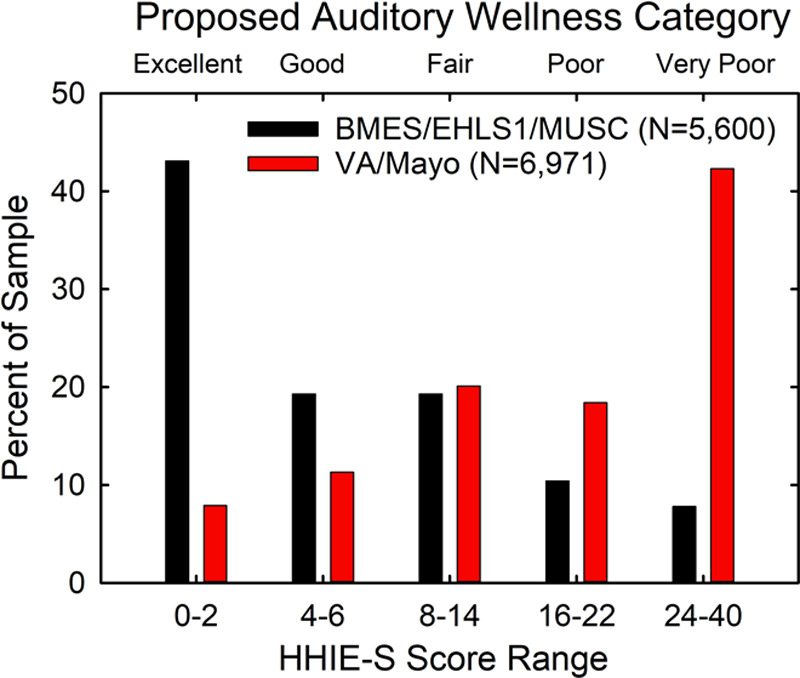
From the equivalent-proportion groupings and likelihood ratios in the lower two panels of Figure 2, auditory wellness ratings or categories were assigned and the distribution of these wellness ratings in each sample type (normative: black; clinical: red) is shown.

Figure 3 shows the percentage of individuals in each of the five auditory wellness categories derived from the HHIE-S scores for the combined normative and clinical datasets (N = 12,571). Clearly, those with “excellent” or “good” auditory wellness ratings most likely came from the nonclinical normative dataset (black bars), whereas those with “poor” or “very poor” wellness ratings likely came from the clinical dataset (red bars). For those in the “fair” auditory wellness category, it is equally likely that their scores are from the nonclinical or clinical datasets. Omnibus Chi-square analysis of these two distributions found a significant difference between the two distributions [X^2^(4) = 3228.7, *p* < 0.001] and follow-up z-tests of the proportions in each category showed that the percentages of each sample differed significantly (*p* < 0.05, Bonferroni-adjusted) between the clinical and normative datasets except for the “fair” auditory wellness category (*p* > 0.05). In terms of the mean HHIE-S scores for each of the five auditory wellness categories, General Linear Model (GLM) analysis showed a significant effect [*F*(4,12,449) = 49,934.2, *p* < 0.001] of wellness category on HHIE-S score and a large effect size (partial Eta-squared) of 0.94. Follow-up Bonferroni-adjusted t-tests showed the mean HHIE-S score for each auditory wellness category differed significantly (*p* < 0.05) from all other categories. Furthermore, when Cohen’s *d* effect sizes were calculated between successive steps on the proposed auditory wellness scale, *d* values exceeded 0.97 for all four comparisons reflecting very large effect sizes (Cohen 1988). Thus, the differences in mean HHIE-S scores comparing those with auditory wellness ratings of “excellent” to “good,” “good” to “fair,” “fair” to “poor,” and “poor” to “very poor” were all large effects. To the extent that establishing likely membership in a nonclinical or clinical population reflects endpoints of an auditory wellness continuum, then the five-point scale based on the HHIE-S appears to be a valid measure of perceived hearing difficulties and, conversely, auditory wellness.

When the HHIE-S scores from each dataset, shown previously in the top panel of Figure 2, were subjected to GLM analyses with age group (50s, 60s, 70s, and 80s) and gender (male and female) as independent variables and better-ear PTA4 as a covariate, the main effects of age group and gender were significant for both the normative dataset [age group: *F*(3,4001) = 1445.9, *p* < 0.001; gender: *F*(1,4001) = 1602.7, *p* < 0.001] and the clinical dataset [age group: *F*(3,6847) = 6002.7, *p* < 0.001; gender: *F*(1,6847) = 2257.6, *p* < 0.001]. The interaction between age group and gender was significant only for the normative dataset [*F*(3,4001) = 171.8, *p* < 0.05]. It should be noted that the normative dataset did not include data from EHLS due to the inability to access the raw data. In addition, there were too few in both datasets to include an age group for those in their 90s. The two datasets were expected to differ in overall HHIE-S scores and this was clearly the case. Furthermore, as confirmed in the GLM analyses of the HHIE-S scores described earlier, significant trends for age group and gender were also apparent. There was a steady decline in HHIE-S score with advancing age for both men and women. Within each age group, women consistently reported less perceived handicap than men. Recall that within each dataset, the better-ear PTA4 served as a covariate. Consequently, the observed effects of age and gender on the HHIE-S scores within each dataset cannot be attributed to differences in hearing loss. Although the EHLS dataset was not included in the normative data here, similar age group and gender effects were observed in those data previously (Wiley et al. 2000), albeit the focus there was on the prevalence of HHIE-S screening failure (HHIE-S > 8) rather than on the HHIE-S scores themselves.

Although there may be no specific expectations regarding the effects of gender on HHIE-S scores, as noted by Wiley et al. (2000), the systematic decline of HHIE-S scores in both datasets with advancing age is somewhat surprising. Wiley et al. (2000) found that several other factors, aside from age, impacted the odds for the self-perception of a hearing handicap, HHIE-S score > 8. They suggested that older adults, here those in their 80s versus those in their 50s, are less bothered by impaired hearing, in the context of other disorders or more restricted social interactions, have fewer demands on their hearing, or have developed better coping strategies.

The motivation for examination of the age group and gender effects centered on the possible development of age- and gender-specific measures of auditory wellness. If there were no obvious effects of either variable on HHIE-S scores, then clearly there would be no need for age- and gender-specific five-point ratings of auditory wellness. The GLM analyses, however, confirm that age and gender *do* impact HHIE-S scores and this is not a secondary consequence of well-known effects of age and gender on hearing thresholds. Future research should examine the possible development of age- or gender-specific scales of auditory wellness as more data become available.

It would be helpful if the proposed five-point scale of auditory wellness based on the HHIE-S could be better validated as a measure of auditory wellness. Unfortunately, large-scale epidemiological studies often are burdened with numerous measures to complete and limited time available to do so. Not only are reliable and valid measures of auditory wellness currently lacking but this is even more true for brief versions of such measures that are practical for large-scale epidemiological studies.

## TRANSLATION FROM HHIE-S TO HHIE-TOTAL

**Fig. 4.**
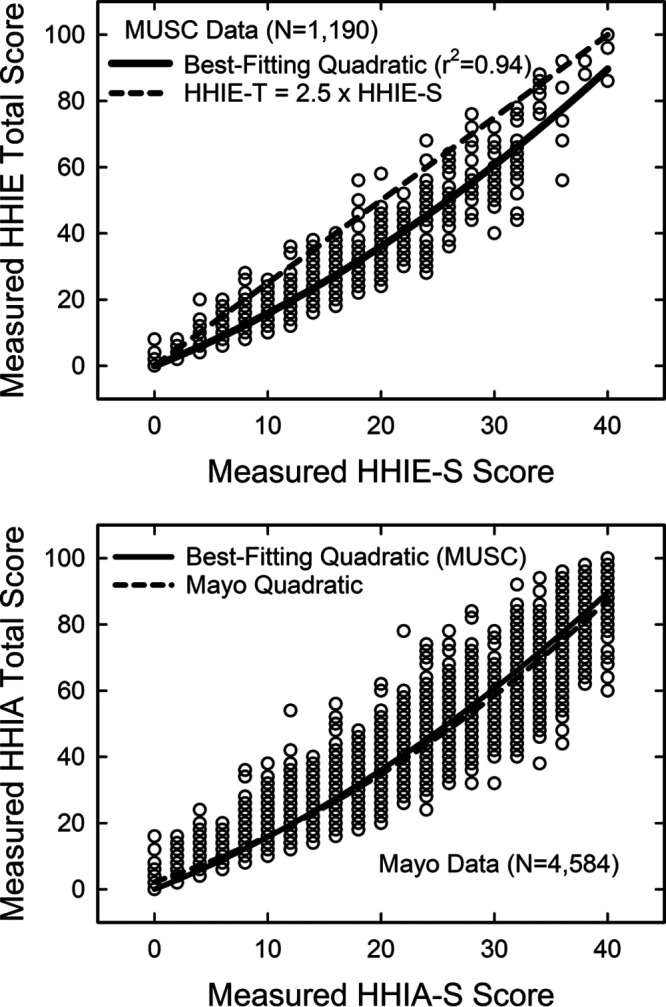
Scatterplots of HHIE-Total scores are plotted against the corresponding HHIE-S scores (top) and HHIA-Total scores plotted against the corresponding HHIA-S scores (bottom) from these same individuals. The top panel shows HHIE data from the MUSC dataset with the best-fitting quadratic equation (solid black line) and the dashed black line in the top panel shows a simple linear relationship between the two scores. The bottom panel shows HHIA data from the Mayo dataset. The best-fitting quadratic function (dashed black line) for the Mayo data and the best-fitting quadratic from the top panel (solid black line) are both shown in the bottom panel. HHIE-S, Hearing Handicap Inventory for the Elderly-Screener.

There are, however, some reasonably sized datasets for the 25-item HHIE-Total measure that allow for further validation against other measures. To transfer the five-point wellness scale derived for HHIE-S scores from thousands of older adults to a similar scale based on HHIE-Total scores, a transfer function must be established. The MUSC dataset included the item responses for the HHIE for 1190 older adults. It was possible to generate both HHIE-S and HHIE-Total scores from these data and the top panel of Figure 4 shows a scatterplot of those scores. The thick black line is the best-fitting quadratic equation which accounted for 94% of the variance (*r*^2^). The dashed black line in the top panel of Figure 4 illustrates a linear relationship between the 40-point HHIE-S and the 100-point HHIE-Total scores. In this case, direct linear equivalency corresponds to simply multiplying the HHIE-S score by 2.5. Although the dashed and solid black lines agree at the zero point, over most of the range, the best-fitting quadratic predicts lower scores than the linear equivalence predicts. The developers of the HHIE-S were aware of this but designed the HHIE-S to have optimum reliability rather than linear equivalence to HHIE-Total scores (Ventry & Weinstein 1983).

The lower panel of Figure 4 provides a scatterplot of data from the Mayo dataset for the HHIA-Total and HHIA-S. As noted previously, the MUSC dataset has data from 160 older adults who completed both tests and those data showed a strong correlation between HHIA and HHIE scores (*r* = 0.995). The best-fitting quadratic (*r*^2^ = 0.915) for the 4584 older adults with HHIA-S and HHIA-Total scores from the Mayo dataset is shown as a dashed black line in the lower panel of Figure 4. Given the equivalence of HHIA and HHIE scores in older adults noted above, it is perhaps not too surprising that there is excellent agreement between the original MUSC transfer quadratic (solid black line in the bottom panel) and the best-fitting quadratic from the Mayo dataset (dashed black line in the bottom panel). The MUSC quadratic was derived directly from HHIE-S and HHIE-Total scores. As a result, this transfer function will be used to convert the five-point scale of auditory wellness based on the HHIE-S to one based on the HHIE-Total. The MUSC transfer function is: HHIE-Total = 0.255 + 1.33 × (HHIE-S) + 0.023 × (HHIE-S)^2^.

**Fig. 5.**
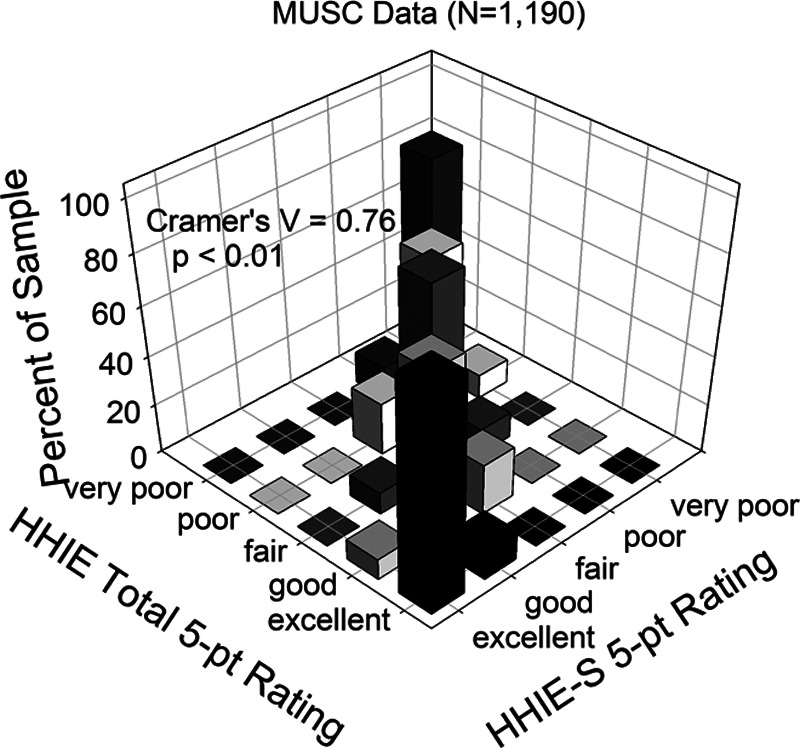
This three-dimensional bar graph provides a graphical illustration of the cross-tabulation of the original HHIE-S-based five-point wellness ratings against the transferred HHIE-Total-based five-point ratings. The percentage agreement of the HHIE-Total ratings with the HHIE-S ratings is plotted for the MUSC dataset. HHIE-S, Hearing Handicap Inventory for the Elderly-Screener; MUSC, Medical University of South Carolina.

Figure 5 illustrates the agreement between the categories of the five-point auditory wellness scale when based on the HHIE-S or the HHIE-Total. There is good agreement between the two five-point scales for 1190 older adults from the MUSC dataset with most of the data falling along the diagonal. This agreement is also supported by a strong Cramer’s *V* (*V* = 0.76; *p* < 0.01).

**Fig. 6.**
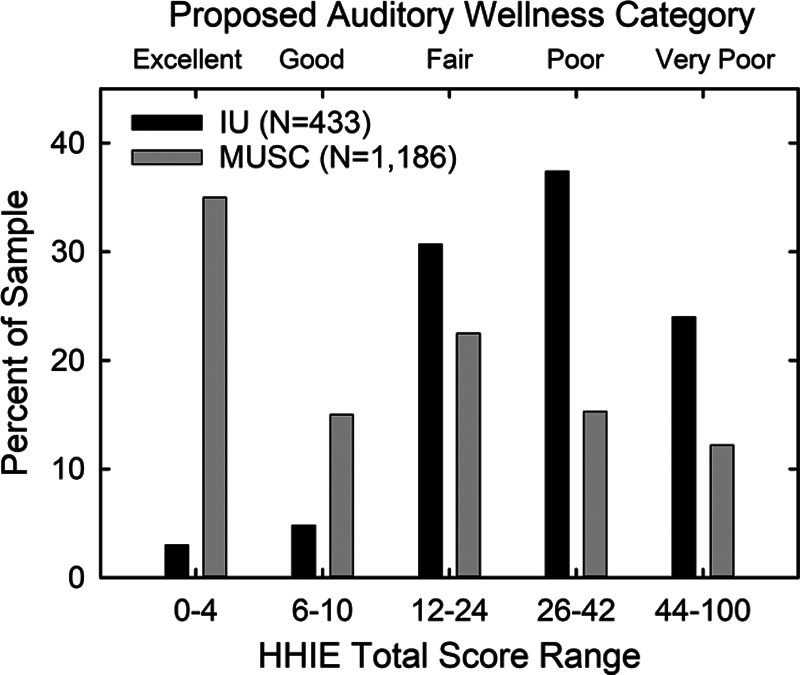
The distribution of proposed auditory wellness ratings based on the HHIE-Total score are shown for a normative sample (MUSC, gray bars) and a clinical sample (IU, black bars). HHIE, Hearing Handicap Inventory for the Elderly; MUSC, Medical University of South Carolina.

Figure 6 shows the distribution of five-point auditory wellness ratings based on the HHIE-Total for the MUSC convenience sample (N = 1186) and a clinical sample from Indiana University (IU; N = 433). The likelihood ratios that would be generated from these data support the validity of the five-point wellness scale based on the HHIE-Total scores, although the segregation of these two sample types (normative versus clinical) is not as clear as that for the five-point scale based on the HHIE-S shown previously in Figure 3. This most likely has to do with the smaller samples involved, a likely mix of population and clinic samples in the MUSC dataset, and the bias of the IU clinical sample toward those with milder amounts of hearing loss than in the larger, broader clinical dataset for the HHIE-S. Chi-square analysis of the distributions in Figure 6 showed a significant difference between the normative and clinical datasets [X^2^(4) = 260.2, *p* < 0.001]. Post-hoc Bonferroni-adjusted z-tests of the proportions showed significant differences (*p* < 0.05) between the two datasets at each of the five auditory wellness ratings. The auditory-wellness category also had a significant effect on the HHIE-Total scores [*F*(4, 1614) = 4039.4, *p* < 0.001] with a large effect size (partial Eta-squared = 0.91). Follow-up Bonferroni-adjusted t-tests showed that the mean HHIE-Total score for each auditory wellness category was significantly (*p* < 0.05) different from those of all other wellness categories. Furthermore, Cohen’s d effect size for each successive paired comparison along the five-point wellness scale exceeded 1.6 for all comparisons indicating very large effect sizes (Cohen 1988) from point to point along the scale. These analyses support the validity of the five-category auditory wellness scale derived from the HHIE-Total scores.

**Fig. 7.**
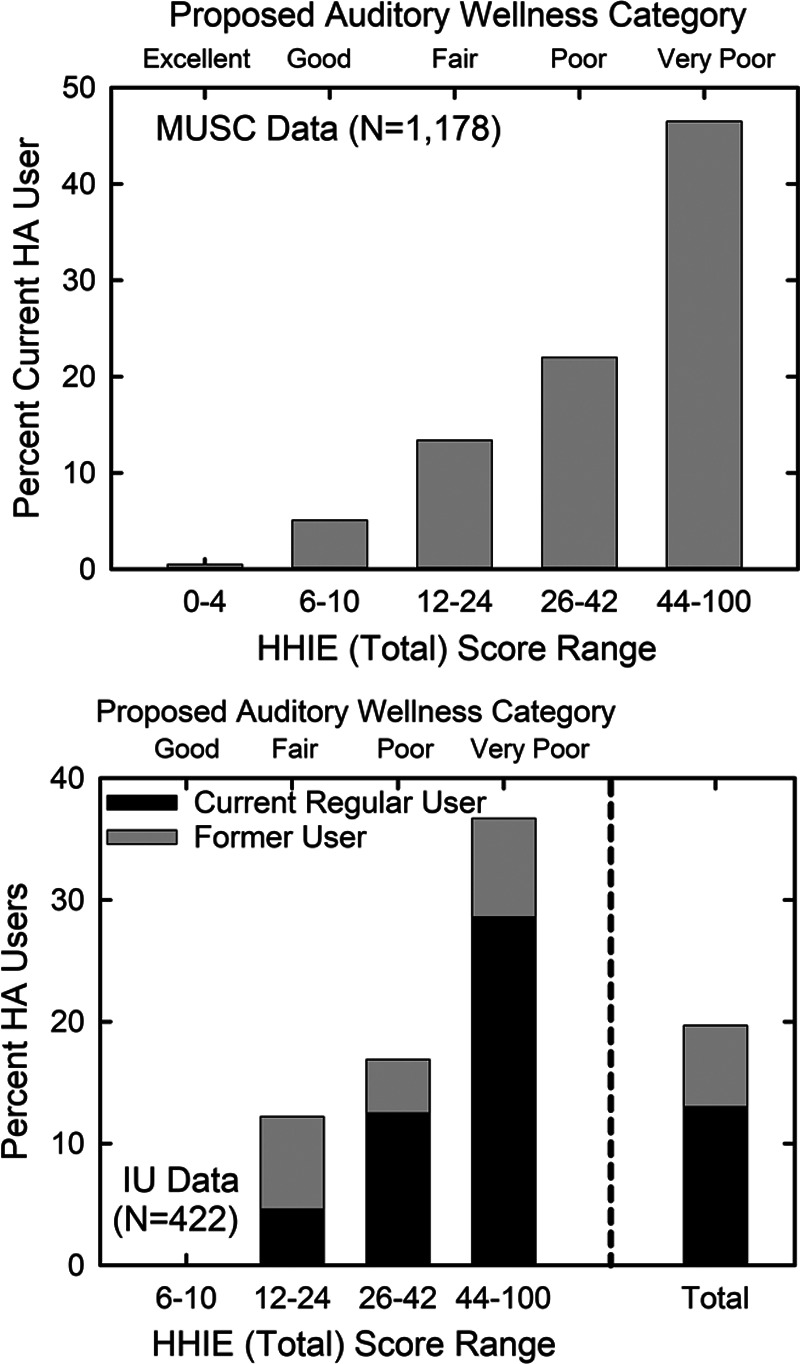
The percentage of hearing aid users within each HHIE-Total-based auditory wellness category are shown for the normative MUSC dataset (top panel) and the clinical IU dataset (bottom panel). In the bottom panel, data were tabulated both for current regular hearing aid use and whether the individual had ever used hearing aids. The bottom panel also shows the total percentages within that sample collapsed across all wellness ratings. Insufficient data were available in the IU dataset to plot data for those with excellent auditory wellness. HHIE, Hearing Handicap Inventory for the Elderly; MUSC, Medical University of South Carolina.

Figure 7 illustrates another approach to evaluation of the validity of the five-point auditory wellness scale based on the HHIE-Total score. Here, hearing aid usage data are plotted for those from the MUSC and IU datasets who responded to a query about hearing aid usage at the time the HHIE was administered. For the MUSC dataset, 152 of the 1178 older adults, or 12.9% were currently using hearing aids. The data in the top panel indicate that the percentage currently using hearing aids increased steadily with the decline in auditory wellness. Few with excellent auditory wellness were current hearing aids users, whereas nearly half of those with very poor auditory wellness wore hearing aids.

For the IU data in the lower panel of Figure 7, more detailed data were available about hearing aid usage at the time the HHIE was administered. Participants were asked whether they had ever worn hearing aids and whether they were currently wearing hearing aids. The combined vertical bars, black + gray bars, reflect the percentage in each auditory wellness category who had ever worn hearing aids. The black bars represent those in each wellness category who were current, experienced, and regular hearing aid users. This category of hearing aid usage was defined as currently using hearing aids, using them for at least 1 year, and wearing them for at least 4 hours daily. For the total group, 20.8% had ever worn hearing aids and 12.9% were current, experienced, and regular hearing aid users. Both categories of hearing aid user increase steadily as auditory wellness declines with 0% of those with good auditory wellness using or having ever tried hearing aids and about one-third of those with very poor auditory wellness using or having ever tried hearing aids. There were too few with excellent auditory health (N = 13) in the IU dataset and this wellness category was omitted here. It is important to note that the percentages for the IU dataset in Figure 7 are for the prevalence of hearing aid usage *before* participation in the IU studies involving hearing aid outcomes. *All* participants in the IU studies responded to ads for participation in studies of hearing aids and were fitted with hearing aids in those studies. Clearly, the IU participants perceived hearing difficulties and sought help via enrollment in these hearing aid studies. Across those studies, all but 15 to 20% retained and used their hearing aids after the 4- to 6-week trial period (Humes et al., 2001, 2017, 2019).

The data on hearing aid uptake and usage in Figure 7 are likely biased toward lower values than one might expect in the future. First, the HHIE was administered and the hearing aid data gathered nearly 20 years ago for many of these participants. This manifest itself in the IU data when 92% of participants reporting current or prior hearing aid usage indicated that they used just one hearing aid. Hearing aid technology and fitting has improved considerably in the intervening years. Another bias is that the gateway to acquisition of hearing aids was controlled by healthcare professionals and candidacy was driven primarily by the audiogram. Thus, it is only those with higher HHIE scores *and* considerable elevation of pure-tone thresholds who would have been considered by healthcare professionals to be candidates for hearing aids. HHIE data from Humes et al. (2017, 2019) were reanalyzed recently based on the severity of pure-tone hearing loss as categorized by the WHO-new HI grade system (Humes 2020a). The average auditory wellness, based on the HHIE-Total, was nearly identical, both unaided and aided, for older adults in the normal, mild, and moderate WHO-new HI categories. When the data in Figure 7 were gathered, most of those in the normal and mild WHO-new HI categories likely would not have been considered strong candidates for hearing aids. Although the trends for increasing hearing aid usage with declining auditory health are accurate and help to validate this measure of auditory wellness, the actual percentages of hearing aid usage in each wellness category will likely rise in the future once direct access to hearing aids is possible.

Another form of validation was possible for subsets of the MUSC and IU datasets. The Communication Profile for the Hearing Impaired (CPHI; Demorest & Erdman 1986, 1987) was obtained from 372 older adults with HHIE-Total scores in the MUSC dataset and 224 such individuals in the IU dataset. The CPHI represents the most comprehensive and rigorously evaluated measure of hearing difficulties and an individual’s reaction to those difficulties. The full CPHI is comprised of 163 items, 25 scales and is ultimately reduced to 5 factor scores. As indicated by Demorest and Erdman (1989a), the three Communication Importance scales of the CPHI are considered extra or optional measures and not a central part of the CPHI which reduces the CPHI to 145 items and 22 scales. Furthermore, two scales of Communication Performance, one for average conditions and one for adverse conditions, are a recalculation of the responses forming three other Communication Performance scales and are excluded due to the redundancy (Demorest and Erdman 1989a). This leaves 20 of the 25 CPHI scale scores that were included in the analyses of the MUSC and IU combined dataset.

The 20 CPHI scale scores from each of the 596 participants were subjected to principal-components factor analysis (Gorsuch 1983). Four factors were identified and accounted for 73.3% of the variance. KMO sampling adequacy index was excellent (0.93) and all communalities exceeded 0.53 with most (13/20) exceeding 0.70. In summary, the four factors identified provided a good description of CPHI performance. In addition, three of the four factors were readily identified as those identified previously by Demorest and Erdman (1989b). These factors were personal adjustment, communication performance, and interaction with others. The fourth factor identified here pertained primarily to the Communication Environment, whereas this was a significant portion of the Reaction factor identified previously by Demorest and Erdman (1989b).

**Fig. 8.**
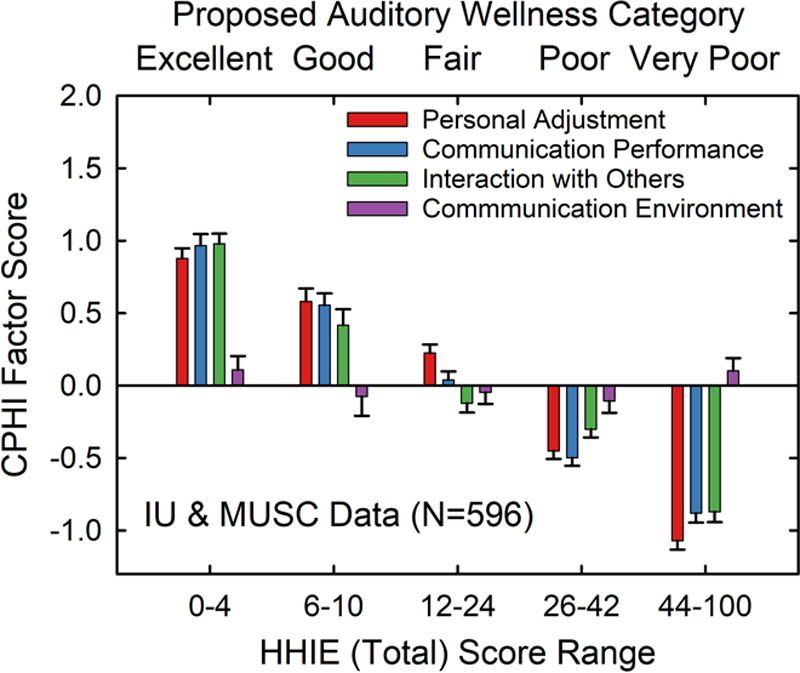
Means and standard errors for CPHI factor scores plotted as a function of the proposed HHIE-Total auditory wellness rating for a combined IU and MUSC dataset (N = 596). Each CPHI factor score is represented by bars of different colors. CPHI, communication profile for the hearing impaired; HHIE, Hearing Handicap Inventory for the Elderly; MUSC, Medical University of South Carolina.

Figure 8 shows the means and standard errors for the four identified CPHI factors scores plotted as a function of HHIE-Total score range or auditory wellness. For three of the four CPHI factor scores, all but the Communication Environment factor score (purple bars), there is a steady decline as auditory wellness based on the HHIE-Total declines. Univariate GLM analyses were conducted with each of the four CPHI factor scores as dependent measures and auditory wellness category as the independent variable. Significant (*p* < 0.01) effects of auditory wellness category on CPHI factor scores were observed for the Personal Adjustment, Communication Performance, and Interaction with Others factor scores, but not for the Communication Environment factor score. For the three CPHI factor scores with significant differences across wellness groups, at least 70% of the paired comparisons were statistically significant (*p* < 0.01, Bonferroni-adjusted). In addition, Cohen’s *d* effect sizes were all large (*d* > 0.66; Cohen 1988) for the paired comparisons of CPHI factor scores representing successive steps along the five-point auditory wellness scale for all four factors. In summary, the group data for this MUSC and IU combined dataset for 596 older adults support the validity of the auditory wellness scale derived from HHIE-Total scores. Communication performance, driven most by hearing loss, declined as auditory wellness declined, but the interaction with others and the individual’s adjustment to impaired hearing also steadily declined as wellness declined.

As noted previously, the HHIE and better-ear PTA4 are moderately and significantly correlated (Fig. 1). For the 596 individuals in the combined MUSC/IU CPHI dataset, the correlation between better-ear PTA4 and HHIE was 0.67. For PTA4 and age, the correlation was 0.37 and for age and HHIE-Total, *r* = 0.17. With an N of 596, all these correlations are statistically significant (*p* < 0.01). It was argued earlier that the HHIE captures the bodily impairment associated with impaired hearing, PTA4, but also is sensitive to the consequences of that bodily impairment on the individual as in the broader WHO-ICF model. Given the significant correlations of HHIE-Total scores with age and hearing loss, especially the latter, perhaps the steady decline in CPHI factor scores with declines in auditory wellness shown previously in Figure 8 are just manifestations of the effects of these other correlated measures (PTA4 and age).

To examine this in more detail, four separate linear multiple-regression analyses were performed, one for each of the four CPHI factor scores derived from the data of the 596 older adults in the combined MUSC/IU dataset. The three independent variables considered were z scores for age, HHIE-Total, and better-ear PTA4. Table 2 summarizes the results. For all four analyses, the VIF and Condition Index values were less than 2.5 indicating no concerns with collinearity among the independent variables. For three of the four CPHI factor scores, the HHIE-Total score is the only one of the three independent variables that was found to be a significant predictor in each of the regression solutions. Moreover, the partial and part correlations were consistently highest for the HHIE-Total when additional predictor variables also emerged. The partial correlation controls for the influence of other independent variables on both the independent and dependent variables. The part correlation, or semipartial correlation, on the other hand, controls for only the associations among the set of independent variables when predicting the dependent variable (Cohen 1988). All three of these regression solutions yielded very large effect sizes according to Cohen’s *f*^2^ effect-size metric for multiple regression [*f*^2^ = *r*^2^/(1 − *r*^2^), unadjusted *r*^2^ values used], with *f*^2^ values of 1.12, 1.11, and 0.71 for the CPHI factor scores of personal adjustment, communication performance, and communication interactions with others, respectively. For the communication environment CPHI factor score regression analysis, *f*^2^ = 0.05, an effect size considered to be small (Cohen 1988).

**TABLE 2.** Regression analyses of four CPHI factor scores with z-transformed age, HHIE-Total score and PTA4 from the better ear as the three independent variables

Dependent Variable						
CPHI PersonalAdjust	Standard β	*t*	Sig.	*r*	Partial *r*	Part *r*
Age (z)	−0.095	−3.12	−0.002*	−0.214	−0.127	−0.088
HHIE-Total (z)	−0.705	−18.52	−0.000*	−0.721	−0.606	−0.522
PTA4 Btr Ear (z)	0.000	0.003	0.998	−0.505	0.000	0.000
CPHI CommPerform						
Age (z)	−0.112	−3.66	−0.000*	−0.293	−0.149	−0.103
HHIE-Total (z)	−0.470	−12.30	0.000*	−0.675	−0.451	−0.348
PTA4 Btr Ear (z)	−0.279	−6.88	0.000*	−0.633	−0.272	−0.195
CPHI InteraxOthers						
Age (z)	0.045	1.33	0.183	−0.121	0.055	0.042
HHIE-Total (z)	−0.471	−11.08	0.000*	−0.621	−0.414	−0.349
PTA4 Btr Ear (z)	−0.237	−5.26	0.000*	−0.535	−0.211	−0.166
CPHI CommEnviron						
Age (z)	0.225	5.179	0.000*	0.217	0.208	0.208
HHIE-Total (z)	0.000	0.004	0.997	0.025	0.000	0.000
PTA4 Btr Ear (z)	−0.020	−0.340	0.734	0.063	−0.014	−0.014

* Significant Beta coefficients, *p* < 0.01. Data from IU and MUSC datasets combined (N = 596).

CPHI, Communication Profile for the Hearing Impaired; HHIE, Hearing Handicap Inventory for the Elderly.

The foregoing analyses of the five-point rating of auditory wellness based on the HHIE-Total score strongly support the validity of this scale. It appropriately captures the sensitivity-loss component of hearing difficulties, resulting in moderate correlations with PTA4, but is also sensitive to the impact of impaired hearing on the person and the person’s adjustment to the impairment. The HHIE-Total score more fully captures the breadth of factors contributing to auditory wellness and conceptualized by the WHO-ICF model of individual function.

Both the HHIE-S and HHIE-Total scores have also been demonstrated to be reliable. For the 10-item HHIE-S, various reliability coefficients have been calculated for sample sizes of 62 (Ventry & Weinstein 1983), 172 (Lichtenstein et al. 1988), and 197 (Tomioka et al. 2013). The reliability coefficients (Kappa, Cronbach’s alpha, Pearson-r test/retest, etc.) have ranged from 0.84 to 0.91. For the 25-item HHIE-Total score, sample sizes have ranged from 16 to 100 with reliability coefficients ranging from 0.84 to 0.96, most commonly >0.90 (Ventry & Weinstein 1982; Weinstein et al. 1986; Newman & Weinstein 1989; Humes et al. 2003). The HHIE-Total has also been used in many studies as a hearing aid outcome measure. The reliability of the aided HHIE scores or the HHIE relative-benefit scores (unaided minus aided HHIE score) is expected to be more variable over time as it not only involves time-varying influences on the person but also on the function of the device as it was worn during the measurement interval. In a series of studies focused on the stability of outcomes over time, the HHIE-Total aided score or the HHIE-Total benefit proved to be stable over measurement intervals from one week to two years and test–retest correlations ranged from 0.70 to 0.95 (Humes et al. 1996, 2002). Given that the proposed auditory wellness scale is based on the HHIE-S or HHIE-Total and these scores have been demonstrated to be reliable over time, the auditory wellness scale derived from these scores is presumed to be reliable as well.

## FUTURE MEASURES OF AUDITORY WELLNESS

The proposed measure of auditory wellness is based on the HHIE, either the full 25-item version or the 10-item screener. Without question the greatest volume of data exists for the HHIE-S. As noted, its brevity is an attractive feature for use in large-scale epidemiological studies. In addition, many of these datasets were established about 10 to 25 years ago and the HHIE-S was one of the few well-developed brief tools available for the self-assessment of hearing difficulties in older adults. In addition, the reliability of the 10-item screener only drops slightly compared to the full 25-item version, with reliability coefficients still exceeding 0.80. The full 25-item HHIE-Total has improved reliability and, as shown above using the CPHI data available from 596 older adults, has excellent validity. Yet, one can question whether the costs in time for the 25-item version are worth the enhanced reliability and demonstrated validity.

Two revised HHIE measures have been proposed recently, each resulting in fewer items while still yielding robust, reliable measures of hearing difficulties and its assumed reciprocal, auditory wellness. In both cases, modern psychometric analyses were used to evaluate the items comprising the 25-item HHIE. Heffernan et al. (2019) used Rasch analysis of the HHIE results from 381 adults who ranged in age from 24 to 90 years (M = 64.5 years, SD = 12.4 years). Given that 80% wore hearing aids every day or sometimes, this is considered a clinical sample. Moreover, the mean HHIE-Total score (unaided) was 45.4 which would place the average score in the “very poor” auditory wellness category, consistent with a clinical sample. In the end, Heffernan et al. (2019) recommended a 16-item improved version of the HHIE.

Cassarly et al. (2020) made use of a nonparametric modern psychophysical method, Mokken scale analysis, to analyze the 25-item HHIE (N = 1064). The mean age for the HHIE-analysis group was 70.4 years (SD = 6.7 years) with a mean *worse-ear* PTA4 of 31.2 dB HL (SD = 15.7 dB). Given this mild hearing loss, only 12.9% in this sample currently used hearing aids, and a mean HHIE-Total score (unaided) of 17.8 (SD = 19.4), this sample is considered a community sample rather than a clinical one like that in Heffernan et al. (2019). After detailed evaluation of the HHIE, a RHHI was developed that consisted of 18 test items common to both the HHIE and the HHIA. The authors, like Heffernan et al. (2019), argued that this revised test was sounder psychometrically than the original 25-item version of the HHIE.

Ideally, in terms of moving forward with HHIE-based measures of auditory wellness, it would be nice if the 16 items identified by Heffernan et al. (2019) were a subset of the 18 identified by Cassarly et al. (2020). Unfortunately, that is not the case. There is some overlap. Eleven of the 25 original HHIE items are common to both proposed revisions. The difference in results of the two analyses using modern psychometric approaches, albeit different approaches, could be due, in part, to the difference in subject samples used. Whereas the MUSC dataset used by Cassarly et al. (2020) represents a convenience sample of the Charleston, SC area, the dataset used by Heffernan et al. (2019) is a clinical sample from the United Kingdom, primarily the Nottingham area of England. It would be ideal to reconcile the differences between the two proposed revisions in the future.

**Fig. 9.**
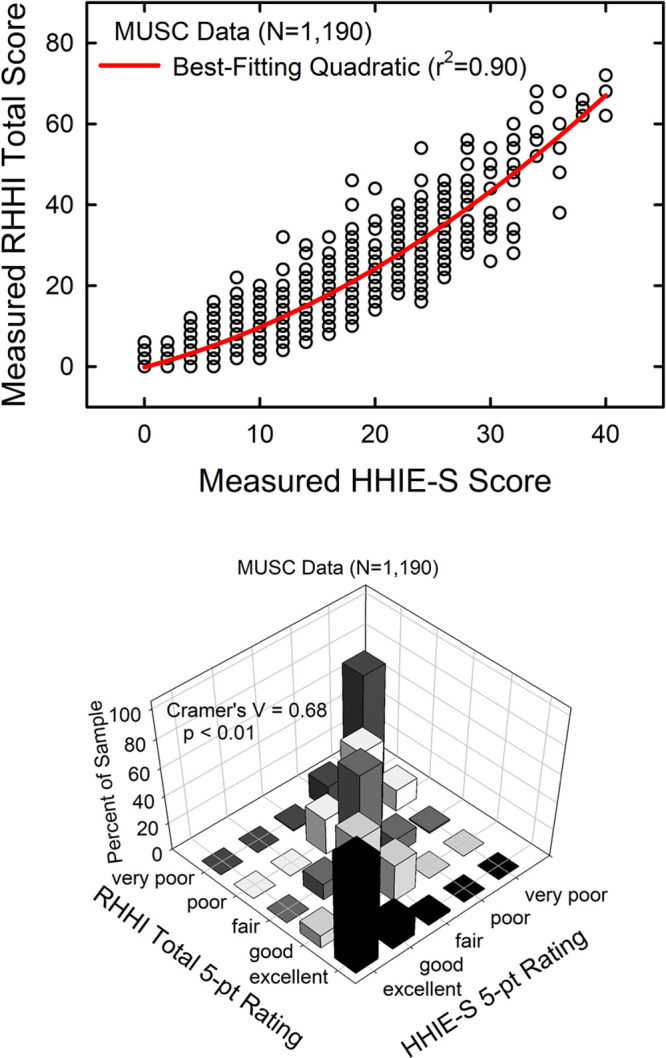
The top panel illustrates the transfer function from the HHIE-S score to the RHHI-Total score for the MUSC dataset. The red solid line is the best-fitting quadratic function. The bottom panel provides a three-dimensional illustration of the cross-tabulation of the two 5-point auditory wellness scales, one based on the HHIE-S and the other on the RHHI-Total scores. HHIE-S, Hearing Handicap Inventory for the Elderly-Screener; MUSC, Medical University of South Carolina; RHHI, Revised Hearing Handicap Inventory.

Due to the nature and size of the MUSC dataset, only the RHHI is considered further here as an updated alternative to the HHIE-based scale of auditory wellness. The correlation between the RHHI-Total and HHIE-Total is 0.99 (*p* < 0.001) for the 1190 older adults in the MUSC dataset. In addition, the correlation of the RHHI-Total with the HHIE-S is 0.93 (*p* < 0.001) for this same dataset. Given that the most robust dataset from which the initial auditory wellness scale was developed was derived from the HHIE-S (Figs. 2 and 3), the relationship between HHIE-S and RHHI-Total scores from the same 1190 older adults was examined. The top panel of Figure 9 shows the resulting scatterplot and best-fitting (*r*^2^ = 0.90) quadratic function (red line). The best-fitting quadratic equation was: RHHI-Total = −0.176 + 0.76 × (HHIE-S) + 0.023 × (HHIE-S)^2^. Application of this function to convert the HHIE-S-based cut points for the proposed five-point auditory wellness scale to RHHI-Total cut points resulted in RHHI scores of 0, 2 to 4, 6 to 16, 18 to 28, and ≥30 for ratings of excellent, good, fair, poor, and very poor, respectively. The bottom panel of Figure 9 shows the agreement between the original HHIE-S auditory wellness categories and the derived RHHI-Total categories. Generally, there is good agreement in the categorization with Cramer’s *V* = 0.68 (*p* < 0.01). Because the RHHI is a subset of 18 of the 25 HHIE survey items, it is most likely that the validation and reliability for the 18-item revised version are the same as the full HHIE-Total, but this should be verified directly.

Although this article has focused on HHIE-based metrics of auditory wellness, due both to the strength of the measure and its widespread use in large-scale studies, it was noted that this is less than ideal because it was not developed to address auditory wellness directly. Rather, the HHIE quantifies whether “a hearing problem” leads to a variety of communication and psychosocial difficulties. As noted earlier, the assumption here is that fewer problems imply better wellness and more problems the opposite. For the CPHI, factor analyses, both our own described above and others by the developers (Demorest & Erdman 1989b), identify 4 to 5 factors, but the two representing most of the variance in those analyses are typically Personal Adjustment and Communication Performance. Recognizing this, and the extraordinary length of the full CPHI, Demorest et al. (2011) described a 20-item screener focusing on just these two factors. For the 596 older adults in the combined MUSC/IU dataset described previously, the correlations between the HHIE-Total score and these two CPHI factor scores, Personal Adjustment and Communication Performance, were −0.72 and −0.68, respectively (*p* < 0.01 for both). The brief version of the CPHI, scored as the original rather than as a pass/fail screener as proposed by Demorest et al. (2011), may hold promise as an alternate measure of auditory wellness. More research is required to evaluate this possibility.

## POSSIBLE IMPLEMENTATION AND APPLICATION

Thus far, three HHIE-based five-point scales of auditory wellness have been described: a 10-item HHIE-S version, an 18-item RHHI-Total measure, and a 25-item HHIE-Total. Various arguments could be made in favor of one version over another. Generally, there is always a tradeoff between test brevity and reliability. This was demonstrated to be true in the prior discussion of the reliability of the HHIE-S versus HHIE-Total but the reliability was still very good for the 10-item screener (*r* > 0.80). Furthermore, the greatest volume of data (e.g., Figs. 1–3) exists for the brief screener. Of course, the time required for completion of the screener is less than half that of the full HHIE, with the RHHI in between. On the other hand, even the full 25-item HHIE takes no more than 10 minutes for older adults to complete. Clearly, there are pros and cons with any of these formats of the HHIE as a potential measure of auditory wellness.

The decision regarding which version of the HHIE to use is not clear at this time. Perhaps, though, a deciding factor may be the intended use of the auditory wellness scale. What is envisioned here is an internet-based online version of one of the HHIE-based scales that could be taken by the older adult at no charge. The survey would be scored immediately online and the respondent would be told his or her auditory wellness rating. Along with labels of “excellent,” “good,” etc., some brief statements might also be included. For example, based on some of the data presented previously in this article, following completion of the online measure, accompanying statements may be included with the score, such as “X% of those over age 50 in the general population had this rating and at least Y% were using hearing aids.” Recommendations might also be made regarding suggested courses of action for each wellness category. Perhaps, for “excellent,” the recommendations may be along the following lines: “you have excellent auditory wellness and it is recommended that you simply re-evaluate your auditory wellness online again in about a year, sooner if you have any concerns.” Whereas, for “poor,” the recommendations might be: “you have poor auditory wellness and would likely benefit from assistance ranging from communication training to the use of hearing aids.”

An advantage in favor of using the full 25-item HHIE is that it could also be used after intervention for a self-administered outcome measure. As noted, the aided HHIE-Total has been proven to be reliable as both an aided-only measure, as well as one of relative benefit (e.g., Humes et al. 2002). Many studies have also documented that the full 25-item HHIE is sensitive to improvements in wellness following intervention with hearing aids (e.g., Weinstein 1990; Humes et al. 1996, 2002; Chisolm et al. 2007) including in the evaluation of hearing aids after self-fitting (Humes et al. 2017, 2019). The volume of evidence regarding its use in documenting effectiveness of intervention is probably the issue that tilts the decision regarding which version of the HHIE to use toward the 25-item HHIE, although it is likely the same results would have been obtained for the 18-item RHHI given the extremely strong correlation between these two measures noted previously (*r* = 0.99). It should also be noted, though, that the shorter HHIE-S has been used as a suitable hearing aid outcome measure too (Newman et al. 1991). It is also because of the envisioned serial use of the online HHIE auditory wellness measure that even slight improvements in reliability, from r values of >0.80 for the HHIE-S to *r* >0.90 for the HHIE-Total, would be the most critical. Critical differences could also be available online for those who use the full HHIE in preintervention and postintervention situations to help the older adult interpret whether a change in wellness over time or after intervention is meaningful. Chisolm et al. (2005), based on unaided and aided HHIE-Total scores from 380 older adults, suggested a 90% critical difference of 15 points for a 2- to 10-week interval between the initial and follow-up HHIE tests. One could also make use of a change in wellness category as a criterion for meaningful change, but the range of scores is nonlinearly mapped to the wellness ratings such that it is possible, especially for those in the “very poor” category at baseline, to make a significant improvement in wellness (reduction in “handicap”), exceeding a 15-point reduction in HHIE-Total score, but not result in a change in wellness rating. Again, more evaluation of the use of the wellness scale in this context is needed. For those failing to achieve a measurable improvement in HHIE score or wellness rating following purchase of a device, supplementary rehabilitation may be recommended, either in person or online. If the device was self-selected, the respondent may also be asked to consider contacting a hearing professional for additional assistance.

It should be noted that others have envisioned the use of either the HHIE-S or HHIE-Total as a part of a software-based aural-rehabilitation system; one that included scores, interpretation of those scores, and added recommendations for intervention (Punch & Weinstein 1996). Holcomb and Punch (2006) further developed and evaluated a “Multimedia Hearing Handicap Inventory (MHHI),” which was delivered via software provided on a compact disc. There were both long and short programs in the MHHI with the short version simply presenting the HHIE-S or HHIE-Total and scoring it automatically. There were provisions to store this information in a local database so that subsequent retakes of the inventory could be compared to earlier results. The long version also included the administration and scoring of the inventory but added additional recommendations and information about hearing difficulties, communication strategies, etc. depending on the outcomes of the testing. Holcomb and Punch (2006) found the MHHI to be both reliable and valid when used with adults.

An online self-administered measure of auditory wellness developed around some version of the HHIE is clearly feasible. As noted, this could be used to guide adults to the correct form of intervention and to also evaluate the benefits of that intervention. Of course, the intervention recommended does not have to be a conventional or OTC hearing aid. Perhaps those with auditory wellness ratings of “good” or “fair,” for example, could benefit from an efficacious group communication-training program (e.g., Hickson et al. 2007) or selective use of assistive listening devices or hearables targeting improvement in the speech-to-noise ratio without providing much gain (e.g., Maidment et al. 2018; Mealings et al. 2020; Singh & Doherty 2020). Further research is needed to map auditory wellness ratings to recommended interventions and then to evaluate the outcomes.

Beginning the candidacy process with a self-report measure of auditory wellness also has the advantage of starting the journey with knowledge of the most predictive factor for hearing aid uptake and success. Individual studies and several literature reviews have repeatedly identified *perceived* hearing difficulties as the key factor for hearing aid uptake and use (e.g., Knudsen et al. 2010; Laplante-Levesque et al. 2012; Hickson et al. 2014; Pronk et al. 2017; Ratanjee-Vanmali et al. 2019; Sawyer et al. 2019; Simpson et al. 2019). Many older adults with slight amounts of pure-tone hearing loss, well within the WHO-new HI range of “normal” hearing, seek hearing aids and obtain positive outcomes (Roup et al. 2018; Humes 2020a; Singh & Doherty 2020). These individuals are already well motivated as they have *perceived* hearing needs that compromise their auditory wellness.

Of course, an auditory-wellness scale is of little or no use if those for whom it has been designed are unaware of its existence. Once an effective auditory wellness scale has been established, partnerships with broad-based organizations such as the American Association of Retired Persons, Consumer Reports, Hearing Loss Association of America, or the NIDCD would be critical to its use. Professional associations also may wish to assist in spreading the word about the availability of such a tool or guide for those with perceived mild-to-moderate hearing difficulties. In addition, although the focus here was placed on older adults with perceived mild-to-moderate hearing difficulties for the reasons noted, there is no reason to restrict the application of a reliable and valid self-administered measure of auditory wellness to just those over the age of 50 years.

In summary, there are many ways in which one could move forward in empowering older adults to manage their own hearing healthcare. The development of valid, reliable tools to assist the older adult in doing so is paramount. A case has been made here for an auditory wellness scale based on some version of the HHIE, a long-standing, well-developed tool. Of course, there are alternate approaches and tools that may be equally valid. The main point, however, is that some good tools need to be widely available soon as the era of OTC hearing aids is dawning in the United States. The worse thing that could happen, regarding improved access and affordability of hearing healthcare, would be to make the process of assessing need and improvement so haphazard that older adults 20 years from now are no better served than they are today. It is important to emphasize, moreover, that this is not envisioned as one model pitted against another with a winner to be crowned. The focus here, as in the OTC Hearing Aid Act, is on adults with *perceived mild-to-moderate* hearing difficulties, the vast majority of whom are 50+ years of age. For those under the age of 18 with any degree of impairment or those adults with more severe hearing loss, the health-professional model has worked well. In addition, for many of those older adults with mild-to-moderate difficulties, additional assistance may be required immediately or eventually to improve auditory wellness. As noted before (Humes 2020b), there is room to incorporate self-directed service provision within existing healthcare profession-driven models to the benefit of all.

## CONCLUSION

This article argues that it is appropriate and critical to move from consideration of age-related hearing loss as a medical disorder diagnosed and managed by others to an auditory wellness model self-managed by the older adult. The older adult should be empowered to self-assess auditory wellness and to respond accordingly to optimize her or his auditory wellness. A self-report scale of auditory wellness that relies heavily on the extensive data available for the HHIE-S was proposed. The proposed measures were translated to HHIE-Total scores and further validated against prevalence of hearing aid usage within each wellness category, by comparisons to results from the CPHI, and by significant improvements in wellness following intervention with hearing aids. More direct measures of auditory wellness may be available in the future, but in the interim, measures based on the HHIE appear to offer valid and reliable options to older adults. An easy-to-use internet-based system of administration, scoring, and interpretation is envisioned as a guide to older adults on their journey to improved auditory wellness.

## ACKNOWLEDGMENTS

The author is indebted to the following individuals who granted access to the de-identified individual data used in this article: B. Gopinath for the Blue Mountains Eye Study (BMES); Richard Wilson for the VA dataset; Judy Dubno for the MUSC dataset; and David Zapala for the Mayo dataset.
